# Case Report: A Novel *de novo* Mutation in DNM1L Presenting With Developmental Delay, Ataxia, and Peripheral Neuropathy

**DOI:** 10.3389/fped.2021.604105

**Published:** 2021-02-26

**Authors:** Yanping Wei, Min Qian

**Affiliations:** Department of Neurology, Peking Union Medical College Hospital, Chinese Academy of Medical Sciences and Peking Union Medical College, Beijing, China

**Keywords:** DNM1L, peripheral neuropathy, dynamin-related protein 1, mutation, MRI

## Abstract

*DNM1L* encodes dynamin-related protein 1 (Drp1), which is a member of the dynamin superfamily of GTPases and mediates mitochondrial and peroxisomal fission. In humans, several *de novo* heterozygous missense mutations in *DNM1L* have been reported, which were characterized by devastating courses with refractory epilepsy, myoclonus, and brain atrophy on MRI. We describe a 4.5-year-old male child harboring a novel *de novo* mutation in *DNM1L* presenting a phenotype of developmental delay, ataxia, and peripheral neuropathy. The clinical features, magnetic resonance imaging findings, and genetic results were summarized. Meanwhile, all the cases of *DNM1L* mutations reported were reviewed. *DNM1L* variants may need to be considered in phenotypes that include global developmental delay, peripheral neuropathy, and ataxia.

## Introduction

Mitochondrial diseases are a group of clinically heterogeneous disorders resulting from mutations in nuclear or mitochondrial genes. Whole-exome sequencing has enabled the identification of nuclear genes affecting mitochondrial dynamics and function. *DNM1L* encodes dynamin-related protein 1 (Drp1), a key component of the mitochondrial fission machinery that is essential for normal mitochondrial function (1). Neurons are particularly vulnerable to Drp1 dysfunction caused by *DNM1L* mutation (2). Mice with constitutive *DNM1L* knockout have smaller forebrains and white matter hypoplasia (2), and most cases of *DNM1L*-related mitochondrial fission defects exhibit refractory epilepsy, myoclonus, and severe global developmental delay (GDD) with a devastating clinical course (3–8). Here, we describe a pediatric case harboring a novel *de novo* mutation in *DNM1L* presenting with a phenotype of developmental delay, ataxia, and peripheral neuropathy.

## Case Description

A male child (currently 4.5 years old) was born at term by vaginal delivery to nonconsanguineous, healthy parents of Chinese ancestry, following a normal pregnancy. Birth weight was 4.0 kg. At 3 months, psychomotor delay was evident, and the child missed motor milestones. At 1.5 years, strabismus was noted that disappeared at 2 years. The child always required assistance to walk. At 4 years, he showed bilateral weakness of the lower limbs and wide-based gait and was prone to falling; he also had difficulty in climbing stairs and rising from the supine position. Fine manipulation ability of both hands was slowed, with abnormal adduction of both thumbs. Meanwhile, mild cognitive impairment was evident. Linguistic function was limited, including dysarthria, and he could only utter non-fluent simple words. Physical examination revealed a body weight of 15 kg and a height of 101 cm. The child was non-dysmorphic but had bilateral everted equinus foot deformity. There were no involuntary movements, and deep tendon reflexes were absent. Medical Research Council strength was 5/5 in both the upper extremities and proximal lower extremities, and 4/5 in plantar and dorsal flexion. Distal muscle atrophy of both legs was detected. Finger–nose–finger and rapid alternating movement tests revealed mild appendicular ataxia. There was no sensory disturbance, and Babinski sign was negative.

## Diagnostic Assessment

**Figure 1:**
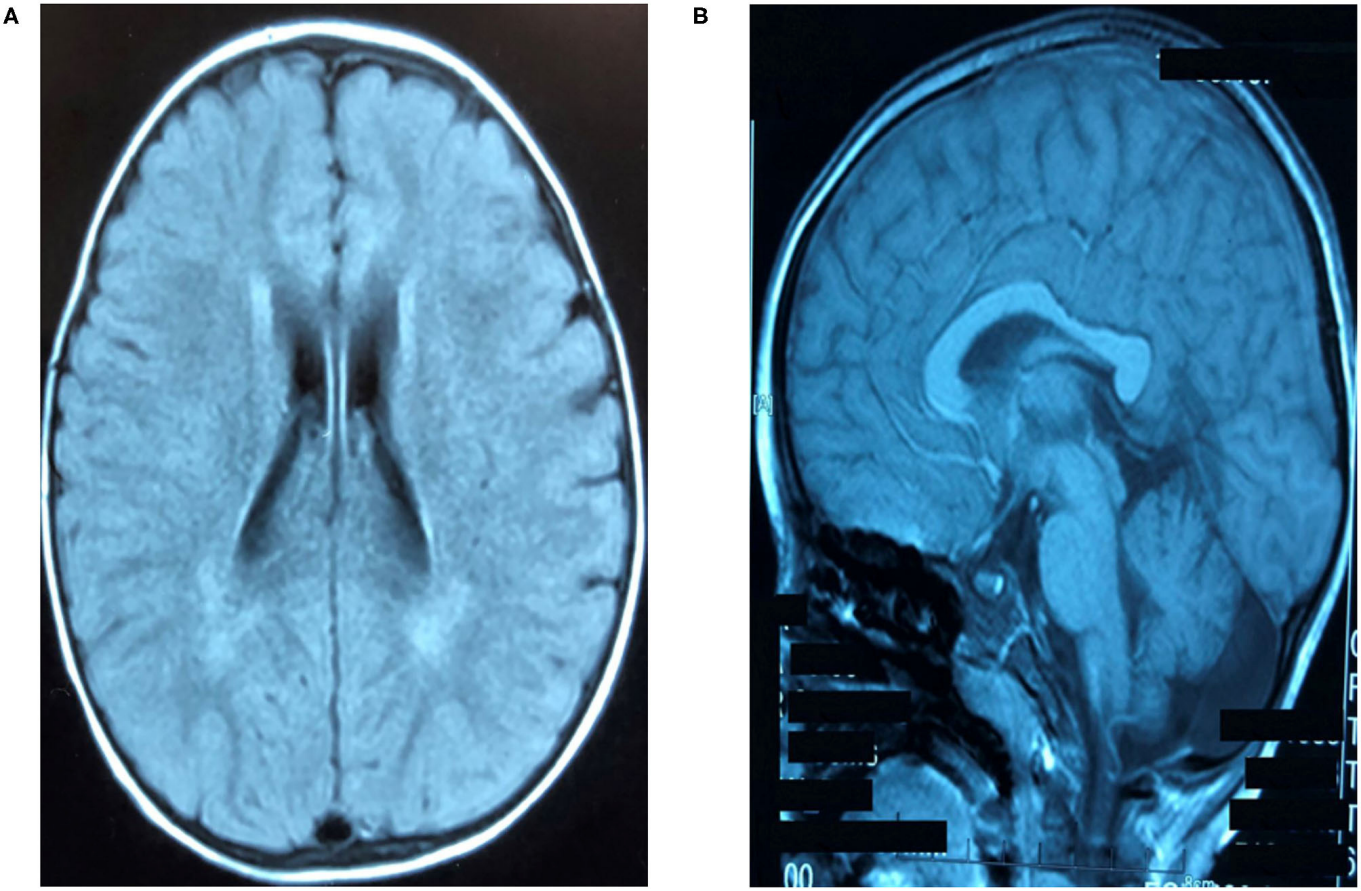
Brain magnetic resonance imaging (MRI) findings. **(A)** Bilateral symmetrical hyperintense lesions of periventricular white matter on T2-weighted images. **(B)** Thinning of the corpus callosum from sagittal view shown on axial T1-weighted images.

The clinical presentation was analyzed and neuroimaging, electrophysiologic, genetic, and metabolic examinations were carried out. The results of blood chemistry and hematologic tests were normal, as were serum amino acid, lactate, pyruvate, and very long-chain fatty acid levels, urine organic acid levels, and acylcarnitine profile in blood spots. Thyroid and liver function were normal. Brainstem auditory evoked potentials and electroencephalogram, electrocardiogram, echocardiography, and ophthalmologic examination results were all normal. Motor nerves of the extremities showed normal functioning. Sensory nerve action potentials (SNAPs) were not elicited in the lower extremities but were markedly reduced in the upper extremities (Table 1). Cranial magnetic resonance imaging (MRI) at 4.5 years of age showed mildly prolonged periventricular white matter relaxation in T2-weighted images, indicating delayed myelination and thinning of the corpus callosum (Figures 1A,B).

Table 1Electrophysiological studies.

**Table:** 

**Motor nerve**	**dl (ms)**	**CMAP amplitude (mv)**	**MNCV (m/s)**
**Right Median nerve**
Wrist-APB	1.6 (<3.8)	10.6 (>9)	51.7 (>50)
Bel Elb-wrist	NT	10.02 (>7)	56 (>55)
**Right Ulnar nerve**
Wrist-ADM	1.4 (<3.0)	8.8 (>8)	NT
Bel Elb-wrist	NT	8.89 (>8)	56 (>55)
**Right Tibial nerve**
Ankle-AHB	2.6 (<4.7)	22.6 (>20)	59.5 (>55)
**Right Peroneal nerve**
Ankle-EDB	2.2 (<3.5)	3.09 (>3)	54.7 (>50)

**Table:** 

**Sensory nerve**	**SNAP amplitude (mv)**	**SNCV (m/s)**
**Right Median nerve**
DigI-Wrist	0.1 (>21)	NT
DigIII-Wrist	0.2 (>9.5)	NT
**Right Ulnar nerve**
DigV-Wrist	1.2 (>7.1)	42.5 (>46.6)
Right Peroneal nerve	NR	NR
Left Peroneal nerve	NR	NR

*dL, distal latency; CMAP, compound motor action potential; MNCV, motor nerve conduction velocity; SNAP, sensory nerve action potential; SNCV, sensory nerve conduction velocity; APB, abductor pollicis brevis; Bel Elb, below elbow; ADM, abductor digiti minimi; AHB, Abductor hallucis brevis; EDB, extensor digitorum brevis; NR, no response; NT not tested; Normal values shown in brackets. Skin temperature was maintained at 36°C*.

We performed whole-exome sequencing of the child and his parents. A missense mutation in the *DNM1L* gene was detected on chr12:32863938 (c.445 G>A, p.G149R). Segregation analysis and Sanger sequencing of family members indicated that this was a *de novo* mutation affecting a conserved glycine (Gly149) in the Drp1 GTPase domain, which is required for the formation of mitochondrial fission complexes and is not known to harbor benign variations according to data in the ClinVar database (http://www.ncbi.nlm.nih.gov/clinvar). This variant was not represented in the Exome Aggregation Consortium (ExAC) database (http://exac.broadinstitute.org/), ExAC of East Asia, Single Nucleotide Polymorphism Database (http://www.ncbi.nlm.nih.gov/snp), or 1,000 Genomes Project (http://browser.1000genomes.org). The global minor allele frequency was <0.005 (http://genetics.bwh.harvard.edu/pph). According to the 2015 American College of Medical Genetics and Genomics guidelines, the mutation is likely pathogenic, which was also predicted by *in silico* algorithms including Sorting Intolerant From Tolerant (http://sift.jcvi.org), Polymorphism Phenotyping v2 (http://genetics.bwh.harvard.edu/pph2), and MutationTaster (http://www.mutationtaster.org).

The child was treated with multiple vitamins including B1, B2, B12, C, E, and coenzyme Q10 taken orally. The patient's condition remained relatively stable without significant improvement in the following 2 years.

## Discussion

**Figure 2:**
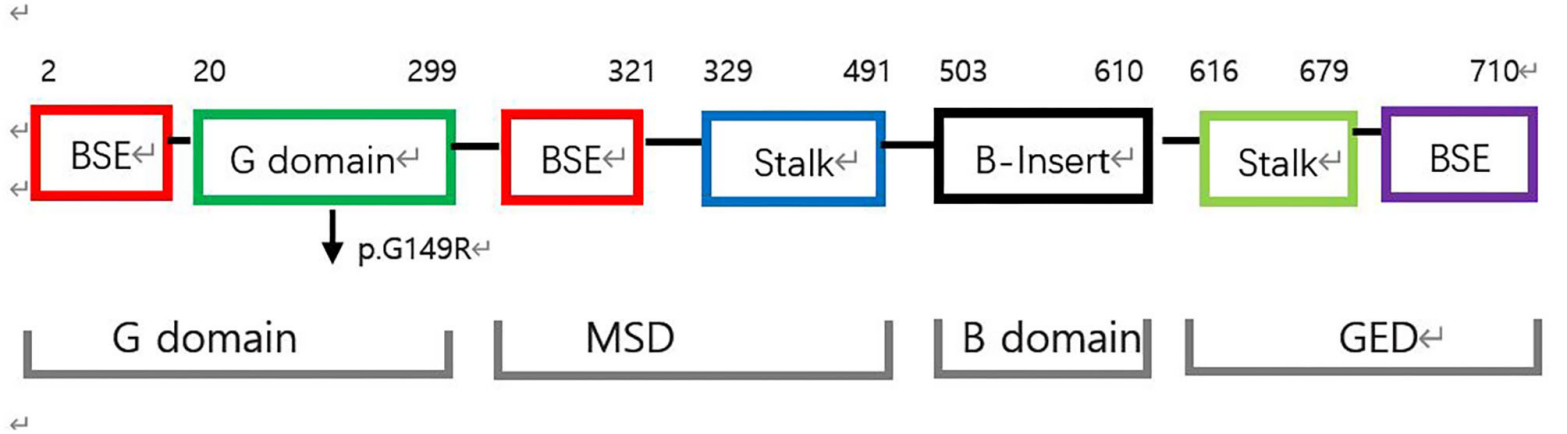
Structure-based domain architecture of human *DNM1L*. The first and last residue of each domain are labeled. The classical predicted domain assignments including the G domain, middle assembly domain (MSD), B domain, and the GTPase effector domain (GED) are shown below. The mutation p.G149R was located at the G domain (black arrow).

**Figure 3:**
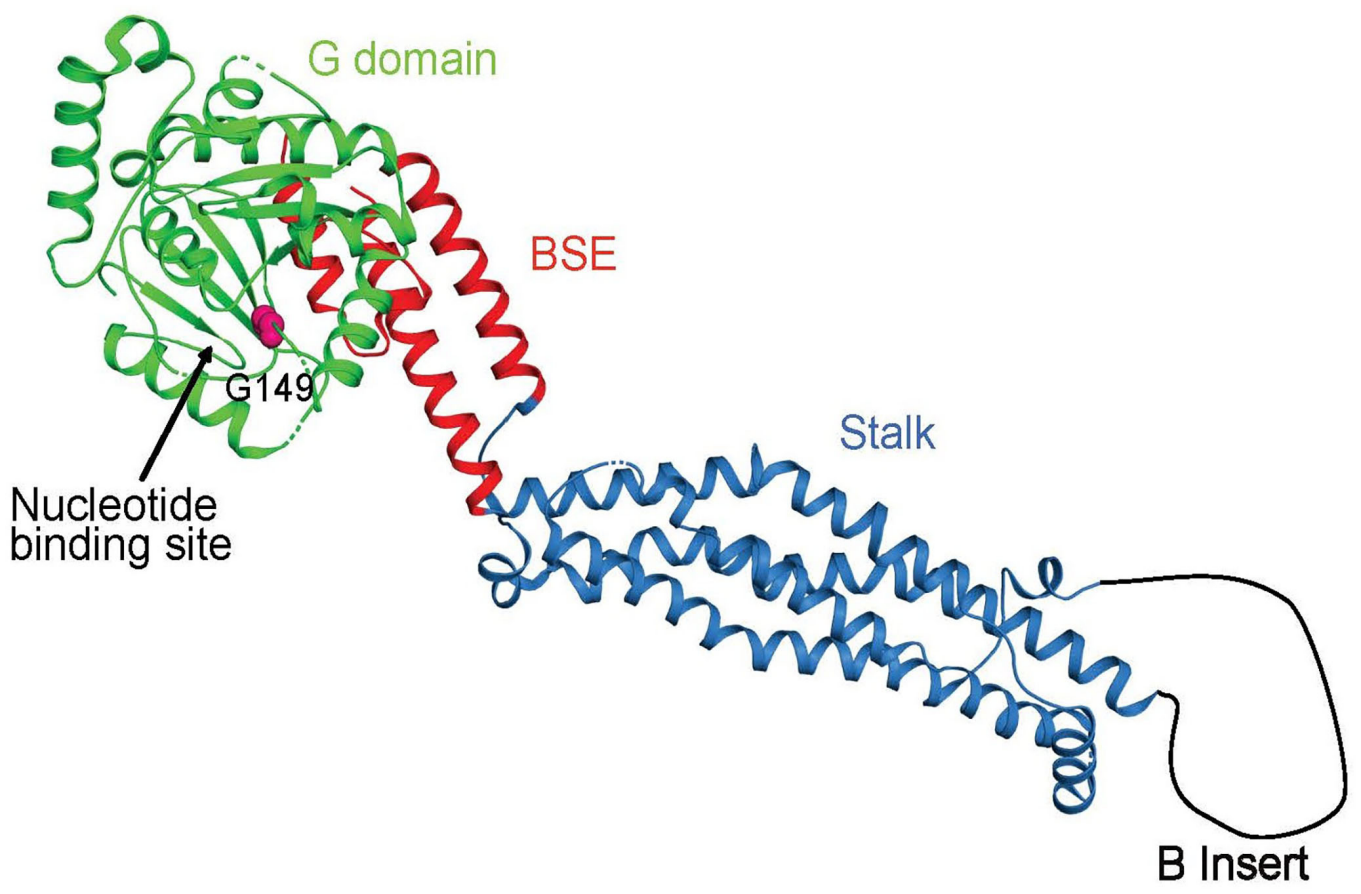
Crystal structure of *DNM1L* in ribbon representation (pdb code 4BEJ). The identified mutation site in the nucleotide-binding cleft is indicated.

Mitochondria continually undergo fusion and fission, which are necessary for their health and normal functioning (1). As a key protein involved in mitochondrial fission, Drp1 is a multimeric dynamin-related GTPase containing an N-terminal GTPase domain (GD) involved in nucleotide binding and hydrolysis; a bundle signaling element (BSE) domain that undergoes nucleotide-dependent conformational changes to mediate motor function; a stalk domain that enables the assembly of Drp1 into helical filaments, and a B domain that is involved in membrane binding and is the site of posttranslational modifications (2–4) (Figures 2, 3). Both the BSE domain and stalk domain contain sequences of the predicted middle assembly domain (MD) and GTPase effector domain (Figure 2). Drp1 is known to oligomerize at the mitochondrial surface into helical filaments that constrict mitochondria to initiate scission. All known point mutations of Drp1 are located in the MD or GD and are associated with specific clinical features ranging from refractory seizures, severe encephalopathy, and even death shortly after birth to peripheral neuropathy, optic atrophy, and late-onset abnormal gait due to spasm, ataxia, or dystonia.

To date, 13 cases of heterozygous *de novo* missense mutations in the MD of Drp1 have been described, with early onset and poor prognosis (5–14) (Table 2). The most frequently detected mutation in the MD was c.1207C>T (p.R403C) (6/13), and the most common clinical features in these cases in order of frequency were as follows: GDD (12/13), refractory seizures and status epilepticus (8/13), hypotonia (3/13), respiratory distress (2/13), and peripheral neuropathy (1/13). Others included dystonia, hemiparesis, ptosis, involuntary movement, lactic acidosis, mild dysmorphic, and optic atrophy (a single case for each symptom). The most common imaging findings associated with MD mutation were diffuse cerebral atrophy, dysmyelination, and abnormal thalamus and brainstem signals.

**Table 2:** Genotype–phenotype imaging correlations among *DNM1L* mutations.

	**Clinical features**	**MRI**	**Num**	**Ref**
**Middle assembly domain**				
c.1207C>T (p.R403C)	GDD, paroxysmal dystonia, RS, SE, myoclonic epilepsy	T2 hyperintensities in thalami, right basal ganglia,bilateral hippocampi	1	(5)
		Brain atrophy		
c.1207C>T (p.R403C)	RS, SE, encephalopathy	Brain atrophy	1	(6)
c.1207C>T (p.R403C)	RS, SE, encephalopathy, GDD	T2 hyperintensity in the thalami, putamen and frontal lobe, DCA	2	(7)
c.1207C>T (p.R403C)	GDD, RS, SE, hemiparesis	T2 hyperintensity in the thalamus, dysmyelination	2	(8)
c.1217T>C (p.L406S)	Hypotonia, infantile spasms, Leigh syndrome, GDD	DCA, diffusion restrictions in the putamen and midbrain	1	(9)
c.1085G>A (p.G362S)	RS, SE, GDD, bilateral ptosis, inverted feet	Normal	1	(10)
c.1084G>A (p.G362S)	Respiratory distress, GDD, insensitivity to pain, athetoid movements, hypotonia	Dysmyelination	1	(11)
c.1084G>A (p.G362S)	Peripheral neuropathy, epileptic encephalopathy,GDD, growth hormone deficiency	Not report	1	(12)
c.1048G>A(p.G350R)	RS, SE, GDD	Dysmyelination, DCA, thinning of CC	1	(13)
c.1135G>A (p.E379K)	Persistent lactic acidosis, apnea, GDD	microcephaly, absence of the CC, DCA, enlarged ventricles	1	(13)
c.1184C>A (p.A395D)	Mildly dysmorphic, hypotonia, absent tendon reflexes, pale optic disc, GDD	Abnormal gyral pattern in both frontal lobes, dysmyelination	1	(14)
**GTPase domain**				
c.261dup (p.W88Mfs*)	Hypotonia, respiratory failure,	Normal	2	(4)
&c.385_386del (p.E129K*6)	low birth weight, peripheral neuropathy	in accordance with hypoxic-ischemic insult		
c.5A>C (p.E2A)	isolated optic atrophy	Normal	13	(15)
c.575C>A (p.A192E)	isolated optic atrophy	Normal	3	(15)
c.106A>G (p.S36G)	GDD, strabismus, dysarthria,	T2 hyperintensities in	2	(16)
&c.346-347delGA (p.E116Kfs*6)	spastic-ataxic gait	subthalamic nucleus		
	GDD, everted equinus foot,	thinning of CC, dysmyelination	1	Ours
c.445 G>A (p.G149R)	peripheral neuropathy, ataxia			

*GDD, global developmental delay; SE, status epilepticus; RS, refractory seizures; DCA, diffuse cerebral atrophy; CC, corpus callosum; Num, numbers of patients; Ref, reference*.

Mutations in the GD have been reported in 20 cases (4, 15, 16), most presenting with optic atrophy (16/20), GDD (2/20), ataxia (2/20), and peripheral neuropathy (2/20). All of the cases had a relatively stable course, except two patients who experienced respiratory failure and died soon after birth (4). Cranial MRI was normal in 18 cases, whereas the other two showed subthalamic lesions or corpus callosum thinning. All reported mutations showed a dominant pattern of inheritance except in four cases of compound heterozygous GD mutations. Thus, GDD is associated with mutations in the MD domain as well as the GD domain of Drp1; seizures are common in cases with MD mutation; and optic neuropathy, ataxia, and peripheral neuropathy are linked to GD mutation.

The main clinical features of our patient were GDD, peripheral neuropathy dominated by sensory nerve lesions, and mild ataxia. Except for optic atrophy, his clinical presentation was similar to that observed in other cases of GD mutation. Although motor nerve conduction velocity was normal, the distal muscle atrophy and weakness suggested motor nerve impairment. SNAPs in the extremities were markedly reduced, indicating severe sensory neuropathy. We identified a *de novo* heterozygous mutation in the GD domain (c.445 G>A) that resulted in a p.G149R mutation in the protein. Gly149 is part of the conserved DxxG motif in the switch II region of Drp1 (3) and participates in the binding of the γ-phosphate of GTP via the main chain. It is thus likely that the p.G149R mutation interferes with GTP binding or hydrolysis and, thus, the motor function of Drp1, resulting in a dominant-negative effect by nonfunctional heteromers formed by the mutated and wild-type Drp1 (3).

Disorders of mitochondrial fusion and fission are a known cause of peripheral neuropathy. Charcot–Marie–Tooth disease 2A2 and hereditary motor and sensory neuropathy VI are caused by mutations in *MFN2* encoding mitofusin 2, a GTPase that mediates the fusion of outer mitochondrial membranes (17). A c.1084G>A missense mutation in the MD was recently described in a patient who presented with sensory and autonomic neuropathy, severe epileptic encephalopathy, and GDD (12).

In this report, we described a case of *DNM1L* mutation and reviewed all known cases in the literature. Patients mostly presented with nonspecific symptoms, which made diagnosis challenging. Whole-exome sequencing may be useful for early diagnosis. Our data suggest that *DNM1L* variants should be considered in the differential diagnosis of hereditary peripheral neuropathy, especially in a background of GDD and ataxia. A limitation of our study is that we could not establish a direct causal link between the p.G149R mutation and altered mitochondrial dynamics; this could be investigated by expressing the Drp1 p.G149R variant in *DNM1L* knockout cells and analyzing the mitochondrial phenotype.

## Data Availability Statement

The raw data supporting the conclusions of this article will be made available by the authors, without undue reservation.

## Ethics Statement

The studies involving human participants were reviewed and approved by Ethics Committee of Peking Union Medical Hospital, Beijing, PR China. Written informed consent to participate in this study was provided by the participants' legal guardian/next of kin.

## Author Contributions

YW made substantial contributions to conception and design, acquisition of data, analysis, and interpretation of data as well as in drafting the manuscript. MQ implied in the interpretation of data as well as gave critical revision of the manuscript. All authors contributed to the article and approved the submitted version.

## Conflict of Interest

The authors declare that the research was conducted in the absence of any commercial or financial relationships that could be construed as a potential conflict of interest.
